# Study on the Curing and Foaming of Surfactant-Modified Geopolymer Gels Based on Ash and Slag Waste from Coal Combustion

**DOI:** 10.3390/gels10010019

**Published:** 2023-12-23

**Authors:** Elena A. Yatsenko, Sergei V. Trofimov, Boris M. Goltsman, Wensheng Li, Victoria A. Smoliy, Anna V. Ryabova, Lyudmila V. Klimova, Andrey I. Izvarin

**Affiliations:** 1 Department “General Chemistry and Technology Silicates”, Platov South-Russian State Polytechnic University (NPI), Prosveshcheniya Street 132, Rostov Region, 346428 Novocherkassk, Russia; e_yatsenko@mail.ru (E.A.Y.); b.goltsman@npi-tu.ru (B.M.G.); vikk-toria@yandex.ru (V.A.S.); annet20002006@yandex.ru (A.V.R.); lyudmila.clim@yandex.ru (L.V.K.); andre.izvarin@yandex.ru (A.I.I.); 2 College of Physics and Electronic Engineering, Northwest Normal University, Lanzhou 730070, China; liws@nwnu.edu.cn

**Keywords:** geopolymer, ash and slag waste, porous structure, surfactant, sodium stearate, microwave curing, the Dumas reaction

## Abstract

This study explores the influence of temperature–time conditions, surfactants, and varied waste compositions on the curing of geopolymer gels, a foam formation with the properties of porous geopolymers. Findings reveal that a 6 h curing period leads to a density of 435 kg/m^3^ and strength of 0.66 MPa, with notable improvements at 12 h. Comparing 12 to 24 h curing, differences in characteristics remain within 5%, highlighting the 12 h period as more energy-efficient. Sodium stearate-based samples exhibit excellent properties, significantly boosting strength while maintaining overall properties. Microwave curing achieves the lowest density (291 kg/m^3^) and closely parallels properties of samples cured conventionally for 12 h. However, it leads to complete destruction in sodium stearate-modified gels due to the Dumas reaction, making it unsuitable above 200 °C. Optimal properties emerge from compositions using sodium stearate and oven curing, achieving densities of 334 kg/m^3^ and strengths of 1.08 MPa (Severodvinsk CHPP-1) and 373 kg/m^3^ and 1.17 MPa (Novocherkassk SDPP). Although microwave curing allows for high energy efficiency, its high temperature demands necessitate careful material selection. This study offers insight into enhancing geopolymer properties while emphasizing the importance of tailored curing methods for sustainable material development.

## 1. Introduction

Currently, environmentally friendly geopolymer materials are gaining increasing popularity worldwide due to their promising combination of physical–chemical properties and low production costs, which is confirmed by a large number of review articles [1,2,3,4,5]. According to J. Davidovits, geopolymers are inorganic materials obtained by alkaline (less frequently acidic [6]) activation of aluminosilicate components present in natural, artificial, or industrial raw materials. The detailed mechanism of geopolymer formation (the geopolymerization reaction) was presented by Rao et al. and Lemougna et al. [7,8].

Depending on the area of application and the required properties, a distinction is made between porous geopolymer materials (geopolymer foam) and geopolymer concrete. Porous geopolymer materials have such promising properties as high porosity (more than 80%) associated with low density (0.30–0.80 g/cm^3^), thermal conductivity (0.05–0.11 W/(m∙K)), and compressive strength (0.2–5.47 MPa) [9,10,11,12]. The potential use of porous geopolymer materials is possible in a fairly wide range, for example, as sorbents for purifying wastewater from chemical and biological pollutants or heavy metals [1,4,13,14], materials for fire protection of construction [2,15,16], catalysts for the synthesis of biodiesel [17,18], as well as in the form of multifunctional coatings for both thermal [7,19] and acoustic insulation [20,21].

Various aluminosilicate raw materials are used for synthesizing geopolymers: natural raw materials include kaolin, volcanic ash, etc. [22,23]; artificial raw materials include metakaolin, metagalluazite, etc. [10,24,25]; and various industrial waste materials include solid fuel residues (ashes, slag, and ash–slag mixtures), rice husk ash, etc. [26,27,28,29,30]. However, it is preferable to use raw materials with a high content of an amorphous phase due to improved reactivity [31]. Utilizing waste as aluminosilicate raw material is more advantageous as it reduces environmental impact while enhancing the economic efficiency of geopolymer production by utilizing easily accessible materials. The most large-capacity among them is ash and slag waste (ASW). According to various estimates, from 750 to 900 million tons of ASW is generated annually around the world [32,33].

The porosity strongly influences the physical and mechanical properties of porous geopolymer materials [2,3,34,35]. Porosity depends on various factors: the type and concentration of foaming and stabilizing additives, as well as the temperature–time curing mode [5]. The most common and easily accessible foaming agents are hydrogen peroxide (H_2_O_2_) with concentrations ranging from 3 to 35% [36,37], and metal powders [38]. Other agents are also utilized: for example, aluminum powder (Al) [39], silicon carbide (SiC) [40], microsilica [41], sodium perborate (NaBO_3_) [42], etc. Foam stabilizers (surfactants) also influence the porosity of the geopolymer and its physical and mechanical properties [35,43]. Different substances are employed as surfactants: palm and sunflower oils [44,45], calcium or sodium stearates [45,46], sodium dodecyl sulfate [47], and others. In review publications [21,35,48], the most common chemical surfactants are calcium stearate and sodium dodecyl sulfate, as they reduce the maximum pore size to 2 mm and improve pore distribution, thereby enhancing the mechanical properties of geopolymers. Among vegetable oils, studies have been conducted with sunflower, palm, olive, and canola oil. Their introduction also allows reducing surface tension. However, the obtained structures are not sufficiently stable and lead to the formation of interconnected pores, distinguishing oils from chemical and commercial surfactants.

Regarding the temperature–time curing modes, low-temperature (20–120 °C [49,50]) and high-temperature treatments (firing) at temperatures of 600–1000 °C [51] are applied. High temperatures positively impact the mechanical properties of products while simultaneously increasing density, thermal conductivity, and energy consumption [52]. Low-temperature curing is more commonly used as it leads to more stable foam formation, positively influencing porosity and density [50].

Microwave curing is less common. Microwaves have the ability to penetrate almost any material and release energy within, heating the entire volume rapidly. Theoretically, this simplifies process control and reduces the cost of synthesizing various materials. Microwaves can be particularly useful for manufacturing porous geopolymers since these materials exhibit low thermal conductivity, and microwave radiation can significantly enhance heat transfer throughout the material. In a series of studies [53,54,55,56], microwave curing was utilized to obtain porous geopolymers without the use of a foaming agent—the foaming occurred due to water evaporation during rapid material heating. Different concentrations of alkali activators from 2 to 15 M (NaOH, KOH) were employed in the investigations, showing improved density results (from 5000 kg/m^3^ to 330 kg/m^3^) with increased concentration. It is noteworthy that in study [54], after microwave curing, they subjected empty coal rock to firing within the temperature range of 500–1100 °C, which does not render the technology energy-efficient. In the study [57], there was also no foaming agent used. However, soybean oil was employed for emulsification and microwave foaming. This method allowed for obtaining porous geopolymers with outstanding results: a density of 287 kg/m^3^ and a strength of 0.45 MPa. These studies confirm that microwave radiation is an innovative method for accelerated heating and, consequently, intensifying the chemical reaction rates.

In the final analysis of articles on microwave curing, no studies were found that examined the combined influence of surfactants of plant and chemical origin with microwave curing. Based on this, the authors set the task of studying the combined effect of microwave curing and surfactants on the formation of the structure and physical–chemical properties of porous geopolymers. This study aims to contribute to the exploration of porous geopolymers as a promising material and in the future, assist in being more attentive to the selection of raw materials and curing mode for synthesizing porous geopolymers.

## 2. Results and Discussion

### 2.1. Optimization of Temperature–Time Mode in the Drying Oven

Preliminarily, the authors investigated curing modes in a drying oven at temperatures of 60, 70, 80, and 90 °C with a duration of 24 h. The optimal mode was found to be at a temperature of 80 °C. It is considered that as the curing time increases, there is an improvement in the ultimate compressive strength of the samples, as the geopolymerization reaction occurs within 20–24 h [58]. However, this mode cannot be considered energy-efficient since the drying oven (at P = 1200 W) consumes approximately 5800 W of electricity over 24 h. Therefore, there was a decision to assess the possibility of optimizing the curing time to reduce energy consumption while maintaining the physical–chemical properties at the same level.

**Figure 1:**
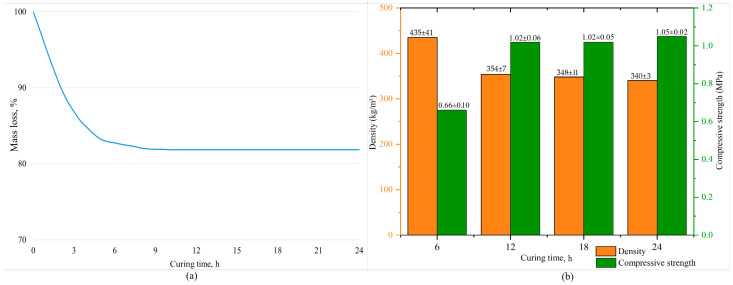
Mass loss (**a**) and physical–chemical properties of porous geopolymers (**b**).

To address this task, the mass loss of the samples was measured over 24 h with a 30 min interval to identify the constant mass point. The mass is considered constant if three consecutive measurements differ by no more than 0.1 mass%. The mass loss is presented in Figure 1a. The investigation of the physical–chemical characteristics (density, ultimate compressive strength) of the samples was conducted every 6 h during curing. The obtained data are depicted in Figure 1b.

The Figure shows that the samples with the maximum curing time have the lowest density (340 ± 3 kg/m^3^) and the highest strength (1.05 ± 0.02 MPa). After 6 h of start of curing, demolded samples were partially destructed. The physical and mechanical characteristics of these samples are at an extremely low level: the compressive strength accounts for 60% of the maximum value, and the density exceeds the minimum by 28%, which is an unsatisfactory result. This is due to the fact that this curing duration is insufficient for the geopolymerization reaction to complete at 80 °C. However, accelerating the process by increasing the temperature could increase the pore volume or lead to the formation of cracks in the geopolymer material, negatively impacting the compressive strength of the samples. This result is in accordance with other studies [59].

Extending the curing time from 6 to 12 h increases the density and strength by an average of 23% and 55%, respectively. With further extension from 12 to 18 h, a slight decrease in density and an average increase in strength of 2% were observed. This trend persisted with the maximum curing time. Ultimately, the difference between 12 and 24 h of curing is no more than 5%, highlighting the 12 h as the most optimal choice for curing time.

### 2.2. Physical–Mechanical Properties of Synthesized Porous Geopolymers

**Figure 2:**
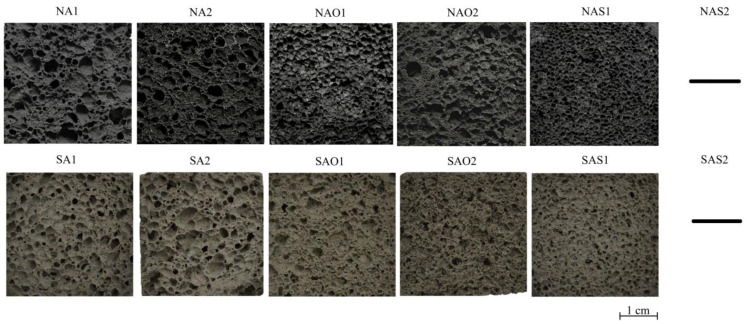
Internal structure of the synthesized porous geopolymers.

**Figure 3:**
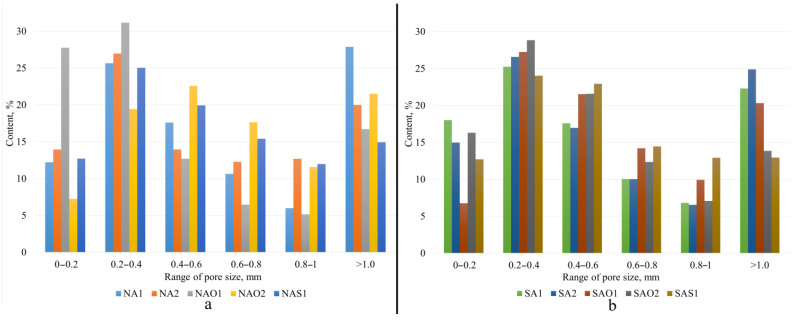
Pore size distribution ranges: (**a**)—Novocherkassk SDPP, (**b**)—Severodvinsk CHPP-1.

The internal structure and physical–mechanical properties of the synthesized samples presented in Figure 2 and Table 1. Figure 3 shows pore size distribution in the samples. The ranges of pore distribution for different compositions are presented in Table 2.

**Figure 4:**
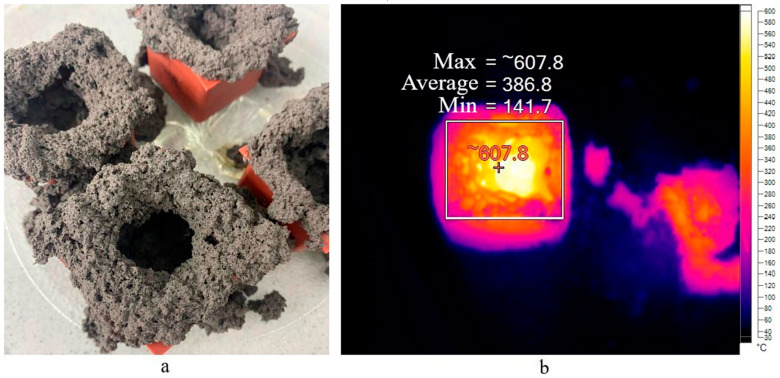
Appearance of destructed samples (**a**) and thermal image of the sample exposed to microwave radiation (**b**).

The absence of a structure for the NAS2 and SAS2 compositions in Figure 2 is explained by the complete destruction of these samples. The result is presented on Figure 4a.

This occurs only with compositions using sodium stearate and microwave curing. After exposure to microwave radiation, the geopolymer was a granular material in the form of cemented particles. Using a thermal imager, it was observed that the outer part of the sample heated to 220 °C (Figure 4b). Simultaneously, the central part heated to 608 °C, a critical point for this composition. A detailed examination of this result is presented in Section 2.4.

According to Figure 2 and Table 1, the use of ASW with different chemical and phase compositions significantly affects the properties of porous geopolymers. Geopolymers based on ASW from Novocherkassk SDPP have, on average, a 12% higher bulk density compared to geopolymers based on ASW from Severodvinsk CHPP-1. One of the influencing factors is the true density of ASW: ASW from Novocherkassk SDPP has a higher true density (2321 kg/m^3^) than ASW from Severodvinsk CHPP-1 (2034 kg/m^3^). However, the difference in the ultimate compressive strength between the wastes is not more than 1.5%.

The curing mode has a lesser impact on the physical–mechanical properties of geopolymers compared to the type of stabilizing additive. Compositions without stabilizing agents (NA1, NA2, SA1, SA2) hereinafter will be described as base compositions. Analyzing compositions NA1 and NA2, it is evident that microwave curing demonstrates better results. Density and strength reduction are at 5% and 6%, respectively. Compositions using olive oil show a similar trend. The strength reduction using microwave radiation was 11% at the same density. This strength reduction is linked to the morphology of foam formation, specifically with pore distribution: composition NAO2 has 50% larger pores (0.38 vs. 0.66 mm). In this study, it was found that unlike the drying oven, microwave curing has a higher material heating temperature, which adversely affected the structure formation with sodium stearate. Additionally, olive oil has a boiling point of 299 °C, increasing the intensity of chaotic foam formation. Therefore, composition NAO2 exhibits numerous large and interconnected pores, close to an oval shape. Ultimately, the combination of these factors deteriorates the strength properties of the composition. Composition NAS1 has enhanced physical–mechanical properties: with a 13% increase in density, the strength increased by 36% compared to the base compositions.

The compositions based on ASW from Severodvinsk CHPP-1 demonstrate a similar trend but with better results. Compositions SA1 and SA2 have the lowest density due to randomly distributed macropores. Compositions using olive oil show a 7% higher density compared to compositions without surfactants. The use of sodium stearate also increased the physical–mechanical properties relative to the base compositions: density and strength increased by 12% and 28%, respectively.

In terms of using surfactants of different origins, sodium stearate appears to be the most optimal. A 3% increase in bulk density correlates with a 42% enhancement in sample strength compared to compositions based on olive oil. 

In general, highly porous material obtained without firing technology always exhibits reduced mechanical properties while maintaining other properties. For instance, in the synthesis of porous geopolymers with acoustic insulation properties, C. Leiva et al. [60] utilized F class fly ash (FA) with the addition of paval waste containing a high content of aluminum oxide (over 75%), using KOH as an alkali, and no foaming agent was applied. Mixing them in various ratios and curing at 70 °C for 24 h resulted in geopolymers with densities ranging from 916 to 1640 kg/m^3^ and strengths from 3.07 to 14.8 MPa. Jiahuan Shao et al. [61] employed bauxite, metakaolin, FA, and NaOH as primary materials, with hydrogen peroxide H_2_O_2_ as a foaming agent. After curing in a closed plastic mold in a drying oven at 35 °C and 75 °C for a total of 48 h, the samples underwent firing at a gradual temperature increase up to 1500 °C for 3 h. Ultimately, porous geopolymers were obtained with densities from 500 to 930 kg/m^3^, strengths from 0.58 to 2.31 MPa, and porosities from 81 to 84%. In the study by Fang Xu et al. [47], similar raw materials (FA, NaOH, H_2_O_2_) were used, varying the initial curing temperature from 25 to 130 °C for 3 h, followed by a 28-day curing period at room temperature. The resulting geopolymers had densities from 500 to 1040 kg/m^3^ and strengths ranging from 3.2 to 44.81 MPa. In the research of Zehua Ji et al. [62], authors used solid wastes (granulated ground blast furnace slag), drinking water treatment residual, and NaOH as primary materials, with H_2_O_2_ as a foaming agent and with surfactants (sodium dodecyl sulfate, sodium alcohol ether sulfate, and disodium laureth sulfosuccinate). Curing in a drying oven at 70 °C for 24 h followed by 7 days at room temperature revealed that sodium dodecyl sulfate was the most effective surfactant. The resulting densities were from 520 to 1330 kg/m^3^, strengths were from 1.76 to 12.14 MPa, and porosities were from 9.94 to 67.89%. In the research by Ehsan Ul Haq et al. [56], fly ash and NaOH (14 mol/L) were used, and the curing of porous geopolymers was conducted using 900 W microwave irradiation for 4 min. Through this study, the authors obtained specimens with densities ranging from 610 to 1300 kg/m^3^, strengths from 3.0 to 6.2 MPa, and porosities from 41 to 72%. The foaming of the geopolymer occurred due to the increased intensity of water evaporation.

The mechanical properties obtained in this study (Table 1) appear to fall within the same range as those reported for porous geopolymers described earlier, and even surpass them in the density values. It is also evident that utilizing a single type of raw material, such as fly ash, does not ensure the stability of physical–mechanical properties due to variations in the chemical composition of the raw material. One of the advantages of this research is the utilization of the most abundant raw materials: ASW generated from solid fuel combustion.

**Figure 5:**
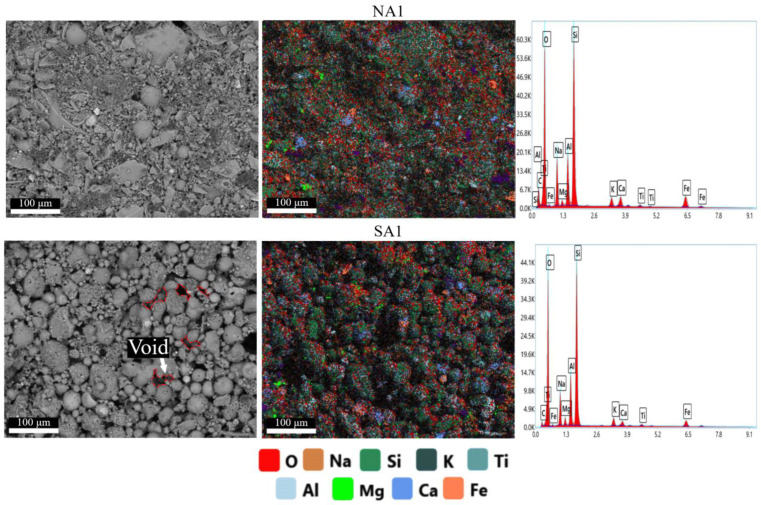
Results of elemental analysis of the surface of porous geopolymers.

Figure 5 shows the mapping results of the surface of geopolymer compositions NA1 and SA1 obtained during EDAX analysis. Images of all synthesized compositions are available in Supplementary Files (Figure S1). The atomic content of elements in all compositions are presented in Table 3.

These results support theoretical calculations of the developed compositions (Section 4.1, Table 5). In addition to light elements such oxygen, the most common elements found in the synthesized geopolymers are silicon, aluminum, and sodium. The use of sodium stearate leads to an increase in sodium content.

Analysis of the obtained SEM images reveals a clear distinction among particles involved in the structure formation of geopolymers. In the NA and NAS compositions, the structure is a conglomerate-type, predominantly composed of angular particles with small agglomerates of rounded particles (close to spherical). These compositions also exhibit a more continuous and dense gel phase with cemented particles, which positively influences the strength of the geopolymer. Conversely, in the SA-SAS compositions, the structure is predominantly represented by spherical or near-spherical particles. Despite a sufficient amount of amorphous gel, these spherical particles have a more porous structure with more voids between them. Their presence reduces the compressive strength and density of the synthesized geopolymers.

The obtained results demonstrate that, besides chemical composition, structure also influences the strength of synthesized geopolymers that is supported by research conducted by other authors [63].

### 2.3. XRD Analysis of Porous Geopolymers

**Figure 6:**
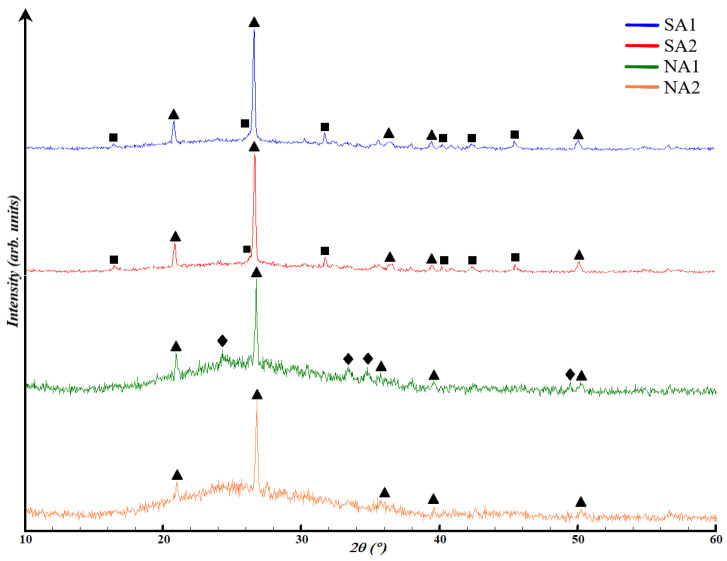
XRD curves of synthesized porous geopolymers: ∆—low quartz (SiO_2_), □—mullite (3Al_2_O_3_ 2SiO_2_), ◊—hematite (Fe_2_O_3_).

The chosen compositions for the structural analysis were the base compositions: SA1, SA2, NA1, and NA2. This decision was made because the X-ray diffractometer only identifies the inorganic component of the material under investigation. Therefore, X-ray diffraction patterns with the application of surfactants would not differ from the base ones. Based on this, Figure 6 presents the results of X-ray phase analysis of porous geopolymers without surfactants. The content of the crystalline and amorphous phase in synthesized porous geopolymers is in Table 4.

All compositions contain both amorphous phase and crystalline phase in the form of quartz, mullite, and hematite. Compositions SA1 and SA2 are almost identical in terms of the crystalline phases appearance and the placement of the amorphous halo in the range of 18–34°. Composition SA1 and SA2 are represented by 59.9 ± 0.6 and 63.9 ± 0.2% of aluminosilicate amorphous phase, and 40.1 ± 0.5 and 36.1 ± 0.7% crystalline phase, respectively. The microwave exposure in composition SA2 increased the content of the amorphous phase by 4%, compared to the previous composition. This result can be explained by the positive impact of temperature on the reaction rate, especially in enhancing the dissolution of the crystalline phase by alkali.

In compositions NA1 and NA2, the amorphous phase is located in the range of 16–38°. Composition NA1 comprises 78.4 ± 0.6% ferroaluminosilicate amorphous phase and 21.6 ± 0.6% crystalline phase. Composition NA2 consists of 84.4 ± 0.4% ferroaluminosilicate amorphous phase and 15.6 ± 0.5% crystalline phase in the form of low quartz. The increase in the amorphous phase content by 14% also occurs due to the influence of high temperature. The reduction of hematite phase can be explained by its interaction with NaOH, according to Equation (1):

Fe_2_O_3_ + 2NaOH → 2NaFeO_2_ + H_2_O (1)

Iron (III) oxide is an amphoteric oxide, which, upon interaction with alkali during heating, forms sodium ferrate (III) with the release of water. A necessary condition for the reaction 1 to occur is heating to a temperature of at least 600 °C. This temperature arises under the influence of microwave radiation with a power of 700 W within the time interval of 120–180 s, confirmed by thermal images of the samples described below (Section 2.4, Figure 10b).

As mentioned below in Section 4.1, ASW is represented by more than 55% amorphous phase. The results obtained show that sodium hydroxide in a 12 M solution partially dissolves quartz and hematite phases. Ultimately, this process increases the content of the amorphous gel from 4 to 14%, thereby improving the reactivity of ASW. Overall, the XRD analysis results are in line with the findings of EDAX and SEM analyses. The increased content of the amorphous gel and the particle shape significantly influence the physical and mechanical properties of porous geopolymers.

### 2.4. Changes in Sodium Stearate during Microwave Exposure

To determine the cause of sample destruction when using sodium stearate, it is necessary to study its interaction with other components. Sodium stearate (NaC_18_H_35_O_2_) is known to be a salt of a carboxylic acid. Carboxylic acids and their salts are characterized by a decarboxylation reaction (elimination) where a molecule of carbon dioxide is released from the carboxyl or carboxylate group. Examples of such reactions include the Kolbe’s reaction, an electrolysis in aqueous solutions of salts, and the Dumas reaction. The Kolbe’s reaction occurs only at electrodes, making it an unsuitable option. The Dumas reaction involves fusing salts of carboxylic acids with alkalis (in our case, sodium hydroxide). The reaction of sodium stearate with sodium hydroxide occurs according to Equation (2):

NaC_18_H_35_O_2_ + NaOH → C_17_H_36_ + Na_2_CO_3_ (2)

As mentioned earlier, decarboxylation involves the cleavage of the carboxylate group (–COONa), resulting in the formation of heptadecane (C_17_H_36_) and sodium carbonate (Na_2_CO_3_), both of which exert negative effects. Heptadecane is an organic compound belonging to the group of alkanes. It possesses low chemical reactivity and can be found in diesel fuel.

**Figure 7:**
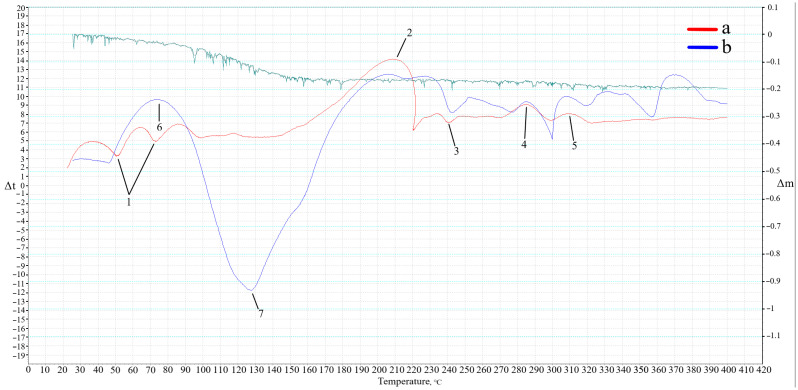
DSC curves of the Dumas reaction (a) and geopolymer gel (b).

It is worth noting that the fusion of sodium stearate with alkali (Equation (2)) occurs only under specific temperatures, which were not found in reference data. Therefore, to identify this temperature and the phase transformations involved, we conducted differential scanning calorimetry using the methodology described in Section 4.3. The DSC curve of the Dumas reaction is presented in Figure 7a. Additionally, to verify the processes occurring during geopolymer synthesis, a DSC analysis of the geopolymer gel of the AS composition was added (Figure 7b).

Analyzing the obtained results of the Dumas reaction, endothermic peaks (1) are observed within the temperature range of 50–100 °C. These peaks are related to the evaporation of physically bound water present in the alkali. The exothermic peak (2) characterizes the reaction temperature according to Equation (2). The initial temperature of the peak is at 145 °C, reaching 221 °C, with an enthalpy of reaction of 45.4 J. The temperature of the peak maximum is at 209 °C, establishing it as the temperature necessary for the reaction to occur. The endothermic peak (3) at 241 °C corresponds to the melting temperature of sodium stearate. Following this, there is an exothermic peak (4) at 287 °C corresponding to the decomposition temperature of sodium stearate within the range of 285–300 °C. The exothermic peak (5) at 305 °C correlates with the boiling temperature of heptadecane.

Examining the DSC results for the geopolymer gel (b), there is partial similarity with the Dumas reaction DSC curve (a), confirming the occurrence of similar processes and phase transformations. However, the intensity of peaks and the sample’s mass change during gradual heating differ noticeably. The endothermic peaks (1) experienced the most significant change. The newly formed exothermic peak (6) corresponds to heat release during the interaction between aluminum powder, alkali, and water, resulting in hydrogen release. The mechanism of this reaction was previously described in the research [26]. The endothermic peak (7) corresponds to the partial dissolution of SiO_2_ and Al_2_O_3_ present in ASW, as well as water evaporation from the gel, confirmed by the mass loss curve. The curve of the mass loss of the geopolymer gel at the temperature corresponding to the maximum of the endothermic peak (7) is 14%.

**Figure 8:**
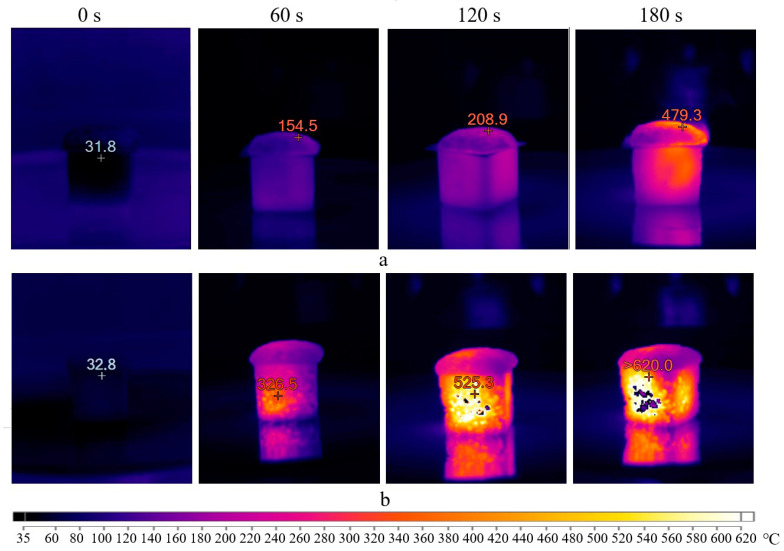
Thermal images of the sample exposed to microwave radiation at 700 W inside molds (**a**) and demolded (**b**).

Based on this, a series of tests were conducted to determine the heating temperature of the porous geopolymer under the influence of microwave radiation for 180 s inside molds and 180 s after demolding. Since sample destruction only occurs when using sodium stearate, the tests used the composition A without surfactants. Initially, microwave radiation at 700 W was applied. The obtained results in the form of thermal images are presented in Figure 8. It is worth noting that the images obtained when placing the sample in the microwave oven (0 s) are displayed in a blue spectrum for simplified visualization. This was performed to view the sample at this stage; otherwise, the first image would be entirely black since (according to the scale used, this shade corresponds to temperatures up to 60 °C).

The initial temperature of the porous geopolymer was 32 °C. After 60 s, the sample in mold (a) was heated up to 155 °C, corresponding to the start temperature of the Dumas reaction. After 120 s, the sample reached 209 °C, signifying the reaction’s completion temperature. At the end of the curing time, the temperature of the sample surface was 480 °C, while the mold temperature exceeded 320 °C. A slight heat flow in the upper right part of the mold was observed after 180 s exposure, attributed to the silicone mold melting.

After cooling, the sample was demolded and placed in the microwave oven. After 60 s, intensive heating of the sample up to 326 °C was observed. This heating intensity was improved by the material’s aggregate state and the absence of the silicone mold. It is known that water evaporation and dissolution of silicates and aluminates in the geopolymer mixture occur during the initial curing. Therefore, after 120 s, the samples inside the molds (a) showed less intense heating, compared to demolded ones (b). At the end of the test, the thermal imager recorded temperatures above 620 °C. In the thermal images, these areas fall beyond the device’s sensitivity range and appear as empty spots.

**Figure 9:**
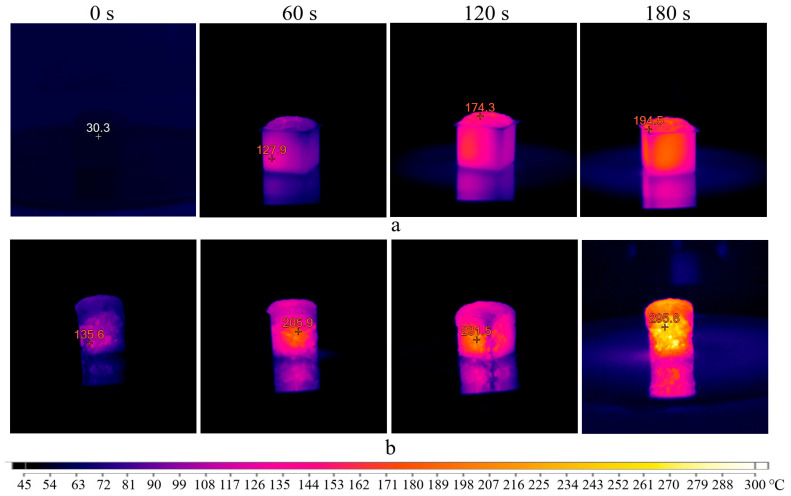
Thermal images of the sample exposed to microwave radiation at 350 W inside molds (**a**) and demolded (**b**).

The microwave oven used has fixed power settings: maximum—100% (700 W), 50% (350 W), and 10% (70 W), respectively. At 10% microwave power for 5 min, the sample did not heat above 40 °C. Consequently, curing under these conditions would take about 15–20 h. Therefore, the second chosen power level was at 50% of the maximum power, corresponding to 350 W. The results are presented in Figure 9.

The real temperature of the samples would be higher than indicated in the thermal images. This is because it is necessary to stop the microwave oven when capturing these images, which stops heating the sample. As a result, the continuous heating of the sample for 180 s would result in a slightly higher temperature. Therefore, this mode also does not correspond to the synthesis of the AS composition. Interestingly, the thermal images obtained reveal the degree of sample heating. Initially, the sample heats from the center to the edges, confirming the theory of microwave penetration. Heating the entire volume take some time. After 180 s, the temperature difference between the central and surface parts of the sample exceeds 100 °C.

**Figure 10:**
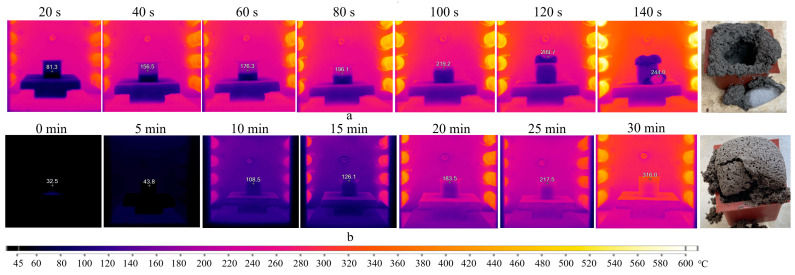
Thermal images of the sample exposed to heating in preheated (**a**) and cold furnace (**b**).

To eliminate the possibility of destructive effects of microwave radiation on the structure of sodium stearate, it was decided that the experiment should be repeated using an electric furnace (TK.8.1300.N.1F, Termokeramika, Moscow, Russia). Two modes were used: the first involved placing the geopolymer gel in the furnace preheated up to 300 °C. The second mode involved gradual heating of the sample at a rate of 10 °C/min to a similar temperature. The thermal images are presented in Figure 10.

The series of thermal images (Figure 10a) depicts a similar pattern as observed in the previous experiments. The mold temperature rises to 157 °C in the first 40 s, indicating uneven heating dynamics of the sample. Subsequently, the process stabilizes, and the rate of temperature increase becomes approximately 1 °C/s. At 80 s, the foaming of the geopolymer gel initiates. The temperature of the mold and gel reaches 196 °C and 140 °C, respectively. Between 120–140 s, the sample undergoes destruction in the temperature range of 140–244 °C.

The mode with uniform heating up to 300 °C (Figure 10b) demonstrates nearly similar outcomes. The exception is the appearance of the sample, which maintains its shape. However, the destruction of the geopolymer occurs when demolding it. This indicates that sodium stearate negatively affects the geopolymer structure not only due to intense heating but also because of the Dumas reaction (Equation (2)), resulting in heptadecane formation.

Summarizing the obtained results, it can be concluded that geopolymers of the AS composition react negatively to both sharp and gradual temperature increases during curing, regardless of the heating source. The melting of sodium stearate with sodium hydroxide occurs at temperatures exceeding 130–140 °C. Therefore, temperatures lower than this range are required for a successful synthesis of porous geopolymers with this surfactant. None of the conducted experiments satisfied these conditions.

## 3. Conclusions

The aim of this work was to study the combined influence of curing conditions, surfactants, and waste with different chemical compositions on the foam formation and the physical–chemical properties of porous geopolymers. The findings can be summarized as follows:

(1) The study of the curing in a drying oven for 6–24 h showed that the difference in physical and mechanical characteristics between 12 and 24 h of curing for the geopolymer gel varied by no more than 5%: the strength increased from 1.02 to 1.05 MPa, while the density decreased from 354 to 340 kg/m^3^. Therefore, a 12 h curing period appears more promising from an energy-efficient standpoint.
(2) Samples containing sodium stearate as a surfactant exhibited the most favorable combination of properties. The following properties were achieved for ASW from different sources: a density of 334 kg/m^3^ and a strength of 1.08 MPa for samples based on the ASW from Severodvinsk CHPP-1, and 373 kg/m^3^ and 1.17 MPa for samples based on the waste from Novocherkassk SDPP, respectively.
(3) The use of microwave radiation allows for achieving the lowest density (291 kg/m^3^) among all samples. The difference between samples’ properties cured using microwave radiation for a total of 6 min and cured using a drying oven at 80 °C for 12 h is almost absent. Thus, microwave curing notably reduces the time of the geopolymer gel curing, ultimately streamlining the production of geopolymers.
(4) However, microwave irradiation adversely affects the sodium stearate-modified geopolymer gels, leading to complete sample destruction due to the formation of the Dumas reaction. Microwave radiation is found to be incompatible with sodium stearate, suggesting the use of this compound at curing temperatures below 200 °C.
(5) Ultimately, the best combination of physical and mechanical properties is exhibited by compositions, utilizing sodium stearate and curing in a drying oven with both types of coal combustion waste. It allows achieving a density of 334 kg/m^3^ and a strength of 1.08 MPa for Severodvinsk CHPP-1 and 373 kg/m^3^ and 1.17 MPa for Novocherkassk GRES, respectively. Microwave curing stands as a promising method for geopolymer material curing in terms of energy efficiency and time savings. Nonetheless, intensive material heating should be considered when selecting raw materials for its production.

## 4. Materials and Methods

### 4.1. Characterization of the Raw Materials

**Figure 11:**
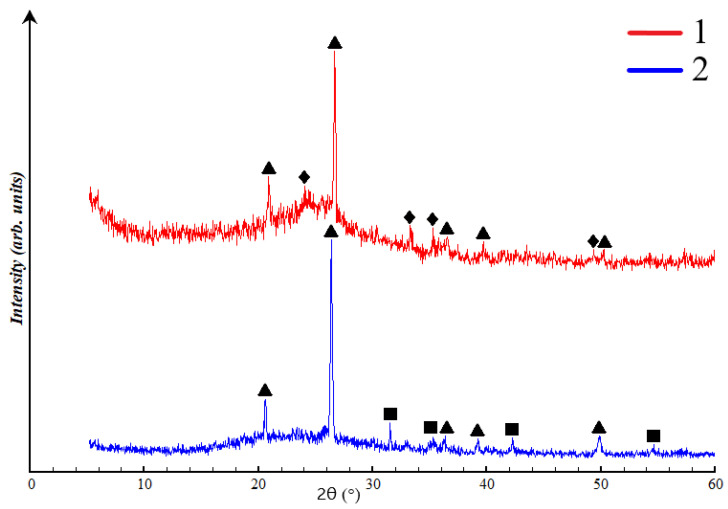
X-ray image of ASW: 1—Novocherkassk SDPP, 2—Severodvinsk CHPP-1, ∆—low quartz (SiO_2_), □—mullite (3Al_2_O_3_ 2SiO_2_), ◊—hematite (Fe_2_O_3_).

To address the stated task, coal-generated ash and slag waste from the Severodvinsk CHPP-1 (Severodvinsk, Arkhangelsk Oblast, Russia) and Novocherkassk State District Power Plant (SDPP) (Novocherkassk, Rostov Oblast, Russia) were utilized as aluminosilicate raw materials. The chemical and X-ray phase composition of the waste is presented in Figure 11 and Table 5. The true density of the waste from Severodvinsk CHPP-1 is 2034 kg/m^3^, while that from Novocherkassk SDPP is 2321 kg/m^3^.

The qualitative X-ray phase composition includes crystalline phases such as low quartz (SiO_2_), mullite (3Al_2_O_3_ 2SiO_2_), and hematite (Fe_2_O_3_), along with the aluminosilicate glassy phase in the diffraction angles 14–38° (2θ) for the Severodvinsk CHPP-1 waste and 18–30° (2θ) for the Novocherkassk SDPP. The quantitative composition comprises 56.9 ± 0.7% amorphous and 43.1 ± 0.8% crystalline structure (32.4 ± 0.5% low quartz and 10.7 ± 0.3% mullite) for the Severodvinsk CHPP-1 waste. The quantitative composition of the Novocherkassk SDPP waste is reported as 70.2 ± 0.7% amorphous phase and 29.7 ± 0.8% crystalline phase (20.4 ± 0.7% low quartz and 9.3 ± 0.1% hematite).

**Figure 12:**
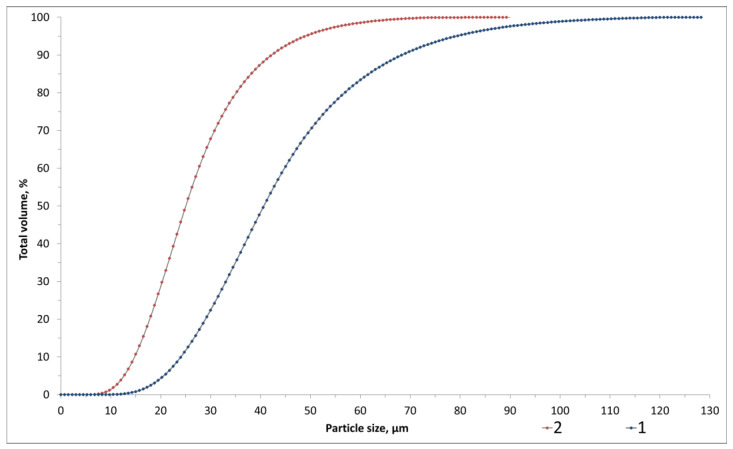
Particle size distribution of ASW: 1—Novocherkassk SDPP, 2—Severodvinsk CHPP-1.

Figure 12 shows the particle size distributions of the ASW, determined using a laser particle size analyzer “LASKA-TD” (BioMedSystem, Saint Petersburg, Russia). The particle size for Severodvinsk CHPP-1 is D50 = 24.94 µm, D99 = 62.59 µm, with a specific surface area of 54.87 m^2^/kg. The particle size for Novocherkassk SDPP is D50 = 40.47 µm, D99 = 101.10 µm, with a specific surface area of 31.94 m^2^/kg.

For alkaline activation of aluminosilicate materials, a mixture of waterglass (sodium hydrosilicate mNa_2_O·nSiO_2_·pH_2_O, silicate modulus = 2, water content 53 wt.%, LONTREK, Moscow, Russia) was used as an activating agent along with a solution of NaOH. As previous studies by the authors have shown [64,65], the optimal molar concentration of NaOH solution ranges from 8 to 12 M, as further increase reduces the compressive strength of foamed geopolymer materials. This conclusion is also supported by research from other authors [66,67]. However, in the study by Xinyu Li [57], the minimum density was achieved at a concentration of 15 mol/L. This indicates that there is no definitive relationship between the concentration of the alkaline activator and the obtained results—each aluminosilicate component requires its specific molar ratio to be determined. Presumably, this effect is achieved due to an increase in the concentration of hydroxide ions that limits their mobility and slows down the formation of coagulation structures [68]. For the preparation of the alkaline solution, a separate container was used, where a pre-weighed portion of granulated NaOH with a purity of 99% (LenReactive, St. Petersburg, Russia) was dissolved in deionized water at a room temperature of 20 ± 1 °C until obtaining a molar concentration of 12 mol/L.

Spherical dispersed aluminum (powder) of ASD-1 grade with a purity of 99% and a specific surface area of 148 m^2^/g (GC Metal Energo Holding, Ekaterinburg, Russia) was used as the foaming agent. Olive oil (CHO Company, Sfax, Tunisia) and sodium stearate (NaC_18_H_35_O_2_, “Standard”, Moscow, Russia) were employed as surfactants (foam stabilizers).

### 4.2. Synthesis of Porous Geopolymers

**Figure 13:**
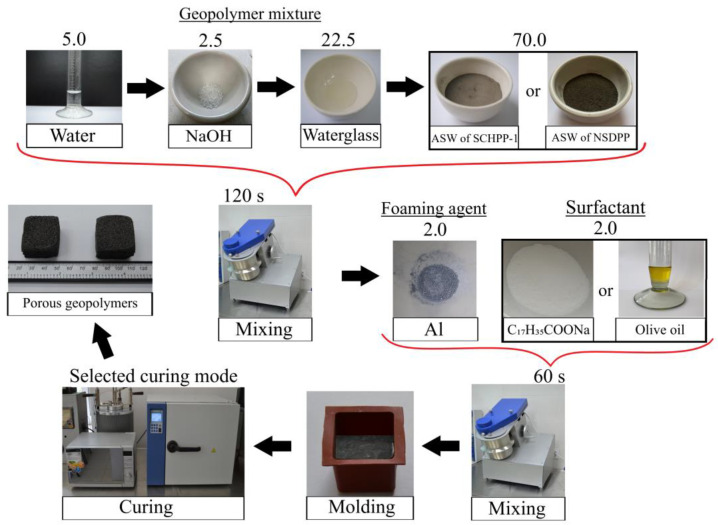
Technology of porous geopolymers’ synthesis.

As mentioned earlier, the precursor used was ash and slag waste from the Severodvinsk CHPP-1 and the Novocherkassk SDPP; the foaming agent—spherical dispersed aluminum, and surfactants—olive oil and sodium stearate. The following notation was used to denote compositions: the coefficient before the composition designates the ASW origin: “N” for Novocherkassk SDPP and “S” for Severodvinsk CHPP-1; “A” stands for base composition using aluminum powder without surfactants; “AO” includes olive oil, and “AS” includes sodium stearate. Digital designations were used to denote curing modes: 1—drying oven, 2—microwave oven. For instance, the designation “SAO2” describes composition using ASW from Severodvinsk CHPP-1, aluminum powder, and olive oil, cured in a microwave oven. The technology for synthesizing porous geopolymers is presented in Figure 13, and the component compositions of the raw mixture are in Table 6.

The technology for synthesizing porous geopolymer materials involved the following sequential stages. Initially, the alkali activator was prepared by the method described in Section 4.1. Then, the obtained gel was poured into a pre-weighed aluminosilicate precursor and mixed for 120 s in a mixer (TL-020, DzerzhinskTechnoMash, Dzershinsk, Nizhny Novgorod region, Russia) at 300 rpm. After mixing, the geopolymer mixture was supplemented with a foaming agent (aluminum powder) and surfactants (olive oil and sodium stearate), followed by an additional 60 s of mixing under the same conditions.

The resulting mixture was poured into silicone cubic molds with a side length of 30 mm for the following curing. A single-stage, low-temperature solidification was used in a drying oven with forced air convection function (LOIP LF 25/350-VS2, LOIP, Saint Petersburg, Russia) at a temperature of 80 °C for 24 and 12 h, respectively. Additionally, the porous geopolymers were cured using microwave radiation at 700 W and 350 W with a frequency of 2500 MHz in a microwave oven (Midea C4E AM720C4E-S, Midea Group Co., Ltd., Foshan, China). A two-stage curing mode was employed: 180 s inside molds and 180 s as demolded samples.

### 4.3. Methods

Qualitative X-ray phase analysis of the synthesized porous geopolymers was conducted using the ARLX’TRA diffractometer (Thermo Fisher Scientific, Waltham, MA, USA) with focused beam reflection via the Bragg–Brentano method. CuKα radiation was employed at 35 kV voltage and with a 30 mA anode current. Diffractograms were recorded in the angular range of 10° to 60° (2θ) with a step size of 0.04°. Data interpretation was carried out using the software package from the ICDD PDF 2 database. Quantitative analysis was performed using the MAUD software (v2.9993 build 532, Materials Analysis Using Diffraction) [69], employing the Rietveld method. The X-ray phase analysis was conducted at the “Nanotechnologies” Collective Use Center of the Platov South-Russian State Polytechnic University (NPI).

Microstructure analysis and EDS (Energy Dispersive Spectroscopy) was conducted using a FEI Quanta 200 scanning electron microscope (FEI Company, Hillsboro, OR, USA). This was coupled with the EDAX Element EDS system (AMETEK, Berwyn, PA, USA). The microanalysis system operated within the following parameters: voltage of 20 kV, magnification (MAG) of 500×, Amp Time (ms): 3.84.

The thermal conductivity of the geopolymers was measured using a thermal conductivity meter (ITP-MG4”100/Zond”, SKB StroyPribor, Chelyabinsk, Russia) employing the stationary heat flow method. The temperature of the hot and cold faces was set at 34 °C and 14 °C, respectively. The device measures the sample height, heat flow density, temperature of opposite faces, and based on the acquired data, calculates the effective thermal conductivity λ, W/m·K, according to Equation (3).

λ = (H·q)/(T_H_ − T_C_), W/m·K (3)

where λ—effective thermal conductivity, W/m·K; H—sample height, cm; q—density of stationary heat flux passing through the measured sample, W/m^2^; T_H_—temperature of the sample’s hot face, K; T_C_—temperature of the sample’s cold face, K.

The methodology and equipment necessary for determining the linear dimensions as well as certain physical–mechanical properties (volume, density, porosity, compressive strength) of porous geopolymers were described in the authors’ previous research [26,64]. Each recorded test value is the medium of 4 measurements.

The pore size and distribution were automatically assessed using ImageJ freeware [70]. Feret’s diameter was used to determine pore sizes, denoting the greatest distance between two points within the pore boundary. High-resolution surface microphotography of samples was conducted using a Stereozoom microscope (SZM-110, The Western Electric & Scientific Works, Haryana, India) equipped with a digital camera (E3ISPM20000KPA IP120000A, Hangzhou ToupTek Photonics Co., Hangzhou, China).

To determine the temperatures of reactions and phase transitions associated with heat release or absorption, differential scanning calorimetry was employed. Analysis was performed using the “Temoskan-2” apparatus (AnalytPribor, St. Petersburg, Russia) with corundum (Al_2_O_3_) used as the reference. Measurements were conducted within a temperature range of 20–400 °C at a heating rate of 10 °C/min. The sample mass used for investigation was 0.5 g.

To determine the surface temperature of the geopolymer, a portable thermal imager (Fluke Ti32, Fluke Corporation, Everett, WA, USA) was used. The temperature was measured by detecting the amount of infrared energy emitted by the sample surface. The device measures temperatures ranging from −20 °C to +600 °C with an accuracy of ±2 °C (but not exceeding 2%). The thermal sensitivity of the device (NETD) at 30 °C is 0.05 °C. Analysis of thermal and visual images was conducted using the built-in software SmartView (v 4.4.363.0, Fluke Thermography, Everett, WA, USA). The emissivity coefficient ε was set at 0.96, which corresponds to concrete. Throughout the study, including the acquisition of thermal images in both the microwave oven and electric furnace, open-type silicone molds were used. To avoid potential damage to the thermal imager, thermal surface imaging of the geopolymer in the microwave oven was conducted with intermittent operation, as indicated in Figure 8 and Figure 9. Hence, the temperatures recorded in Figure 8 and Figure 9 will be lower than those obtained under continuous microwave exposure for 180 s. To prevent significant heat loss during the cold electric furnace test, the door was closed. The door was opened and imaging was conducted at 5-min intervals. During the acquisition of thermal images in the preheated furnace, the door remained open throughout the experiment. The imaging frequency was limited by the technical capabilities of the thermal imager: specifically, the speed of recording thermal images on the SD memory card (1 frame every 10–12 s) and the automatic calibration of the infrared lens (3–5 s), collectively determining the imaging rate of 1 thermal image per 12–15 s.

## Figures and Tables

**Table 1:** Medium physical–mechanical properties of the synthesized porous geopolymers.

#	Density, kg/m^3^	Compressive Strength, MPa	Porosity, %	Thermal Conductivity, W/(m·K)
NA1	339 ± 11	0.88 ± 0.05	85.8 ± 0.2	0.0754 ± 0.0038
NA2	322 ± 15	0.83 ± 0.03	87.7 ± 0.7	0.0719 ± 0.0031
NAO1	364 ± 14	0.86 ± 0.02	84.8 ± 0.8	0.0750 ± 0.0031
NAO2	361 ± 18	0.77 ± 0.06	84.9 ± 0.9	0.0800 ± 0.0009
NAS1	373 ± 7	1.17 ± 0.08	84.3 ± 1.0	0.0826 ± 0.0018
SA1	306 ± 3	0.85 ± 0.03	85.0 ± 0.1	0.0700 ± 0.0005
SA2	291 ± 5	0.83 ± 0.03	85.7 ± 0.7	0.0670 ± 0.0008
SAO1	326 ± 4	0.78 ± 0.03	84.0 ± 0.2	0.0745 ± 0.0009
SAO2	322 ± 9	0.73 ± 0.02	84.1 ± 0.9	0.0736 ± 0.0014
SAS1	334 ± 11	1.08 ± 0.03	83.6 ± 0.6	0.0761 ± 0.0021

**Table 2:** The pore distribution of the synthesized porous geopolymers.

#	D50	D90	D99
NA1	0.53 ± 0.07	1.97 ± 0.03	4.24 ± 0.21
NA2	0.56 ± 0.08	1.69 ± 0.07	3.94 ± 0.17
NAO1	0.38 ± 0.03	1.48 ± 0.03	3.61 ± 0.24
NAO2	0.66 ± 0.05	1.59 ± 0.05	2.87 ± 0.18
NAS1	0.48 ± 0.04	1.34 ± 0.05	2.49 ± 0.13
SA1	0.43 ± 0.04	1.67 ± 0.06	3.53 ± 0.17
SA2	0.52 ± 0.07	1.90 ± 0.11	4.95 ± 0.21
SAO1	0.55 ± 0.05	1.17 ± 0.06	2.05 ± 0.22
SAO2	0.48 ± 0.04	1.22 ± 0.05	2.60 ± 0.22
SAS1	0.51 ± 0.04	1.34 ± 0.03	2.59 ± 0.11

**Table 3:** Content of elements in the synthesized samples, atomic%.

Composition	O	Na	Al	Mg	Si	K	Ca	Ti	Fe	∑
NA1	59.9	11.2	5.8	0.9	16.5	1.0	1.3	0.2	3.2	100.0
NA2	57.1	12.3	6.1	1.1	18.3	1.2	1.4	0.3	2.2	100.0
NAO1	58.3	13.7	5.6	1.0	16.7	1.1	1.4	–	2.2	100.0
NAO2	60.4	12.8	5.2	1.0	16.1	1.0	1.2	0.2	2.1	100.0
NAS1	57.1	16.7	5.1	0.9	15.9	1.0	1.2	0.2	1.9	100.0
SA1	62.1	8.4	6.2	1.4	18.3	1.0	0.8	0.3	1.5	100.0
SA2	62.4	10.9	5.6	1.3	16.9	0.9	0.6	0.2	1.2	100.0
SAO1	62.2	9.1	5.9	1.4	17.5	1.0	0.9	0.2	1.8	100.0
SAO2	62.0	9.2	6.1	1.4	17.9	1.0	0.7	0.3	1.4	100.0
SAS1	62.0	13.7	4.9	1.4	15.5	0.7	0.6	0.2	1.0	100.0

**Table 4:** Content of crystalline and amorphous phase in synthesized porous geopolymers, wt.%.

	Low Quartz	Mullite	Hematite	Amorphous Phase
NA1	13.6 ± 0.5	–	8.0 ± 0.1	78.4 ± 0.6
NA2	15.6 ± 0.5	–	–	84.4 ± 0.4
SA1	31.3 ± 0.4	8.8 ± 0.1	–	59.9 ± 0.6
SA2	29.1 ± 0.6	7.0 ± 0.1	–	63.9 ± 0.2

**Table 5:** Chemical composition of raw materials, wt.%.

Component	SiO_2_	Al_2_O_3_	Fe_2_O_3_	MgO	Na_2_O	K_2_O	CaO	TiO_2_	MnO	P_2_O_5_	SO_3_	LOI
ASW from Severodvinsk CHPP-1	61.6	17.9	6.0	2.7	3.6	2.3	2.1	0.8	0.1	0.2	0.3	2.3
ASW from Novocherkassk SDPP	51.2	18.8	10.3	2.1	0.9	3.0	3.1	0.8	0.1	0.1	0.3	9.2
Waterglass	29.8	0.6	0.1	–	15.3	–	0.2	–	–	–	0.2	53.8
Geopolymer based on ASW from Severodvinsk CHPP-1	49.7	12.7	4.2	1.9	7.7	1.6	1.5	0.6	0.1	0.2	0.3	19.5
Geopolymer based on ASW from Novocherkassk SDPP	42.4	13.3	7.2	1.5	5.8	2.1	2.2	0.6	0.1	0.1	6.5	18.2

**Table 6:** Compositions of studied porous geopolymer mixtures, wt.%.

#	Precursor (ASW)	NaOH (Powder)	Water	Waterglass	Aluminum Powder, over 100	Olive Oil, over 100	Sodium Stearate, over 100
A	70.0	2.5	5.0	22.5	2.0	–	–
AO	70.0	2.5	5.0	22.5	2.0	2.0	–
AS	70.0	2.5	5.0	22.5	2.0	–	2.0

## Data Availability

Publicly available datasets were used in this study. This data can be found in the cited references. All data presented in this study are included in the published article.

## Supplementary Materials

The following supporting information can be downloaded at: https://www.mdpi.com/article/10.3390/gels10010019/s1, Figure S1: Elemental analysis of the surface of porous geopolymers.
