# Supplementary figures and images for: Study on the Curing and Foaming of Surfactant-Modified Geopolymer Gels Based on Ash and Slag Waste from Coal Combustion
Source: Gels. 2023 Dec 23;10(1):19. doi: 10.3390/gels10010019 (PMC10815204; doi:10.3390/gels10010019)

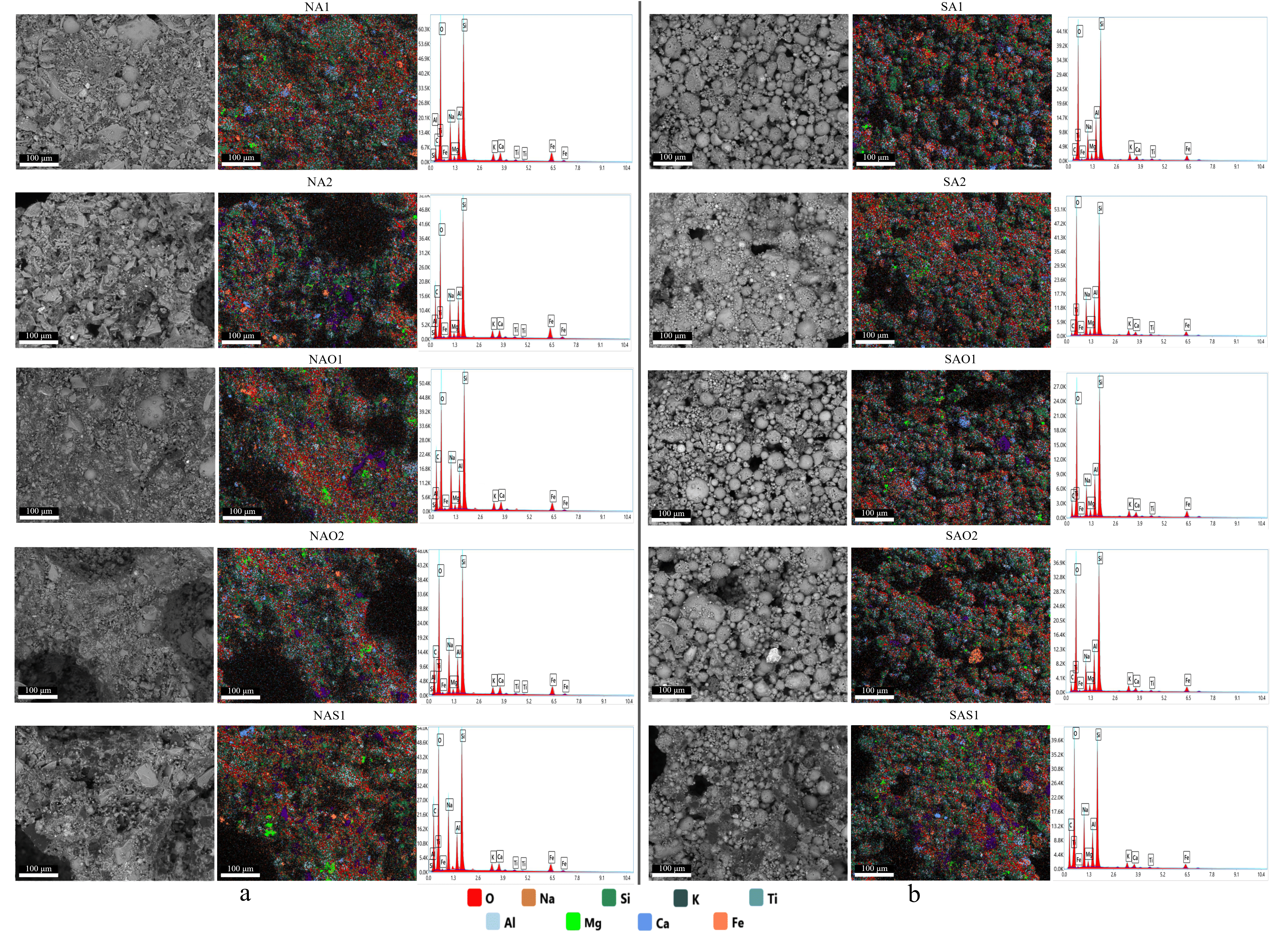

Supplement: Supplementary file 1 [file gels-10-00019-s001.zip › Figure S1 Elemental analysis of the surface of porous geopolymers.png]
